# Systematic Expression Profiling Analysis Identifies Specific MicroRNA-Gene Interactions that May Differentiate between Active and Latent Tuberculosis Infection

**DOI:** 10.1155/2014/895179

**Published:** 2014-09-04

**Authors:** Lawrence Shih-Hsin Wu, Shih-Wei Lee, Kai-Yao Huang, Tzong-Yi Lee, Paul Wei-Che Hsu, Julia Tzu-Ya Weng

**Affiliations:** ^1^Institute of Medical Sciences, Tzu Chi University, Hualien 97004, Taiwan; ^2^Taoyuan General Hospital, Ministry of Health and Welfare, Taoyuan 33004, Taiwan; ^3^Department of Life Sciences, National Central University, Taoyuan 32001, Taiwan; ^4^Department of Computer Science and Engineering, Yuan Ze University, Taoyuan 32003, Taiwan; ^5^Innovation Center for Big Data and Digital Convergence, Yuan Ze University, Taoyuan 32003, Taiwan; ^6^Bioinformatics Core Laboratory, Institute of Molecular Biology, Academia Sinica, Taipei 11529, Taiwan

## Abstract

Tuberculosis (TB) is the second most common cause of death from infectious diseases. About 90% of those infected are asymptomatic—the so-called latent TB infections (LTBI), with a 10% lifetime chance of progressing to active TB. To further understand the molecular pathogenesis of TB, several molecular studies have attempted to compare the expression profiles between healthy controls and active TB or LTBI patients. However, the results vary due to diverse genetic backgrounds and study designs and the inherent complexity of the disease process. Thus, developing a sensitive and efficient method for the detection of LTBI is both crucial and challenging. For the present study, we performed a systematic analysis of the gene and microRNA profiles of healthy individuals versus those affected with TB or LTBI. Combined with a series of in silico analysis utilizing publicly available microRNA knowledge bases and published literature data, we have uncovered several microRNA-gene interactions that specifically target both the blood and lungs. Some of these molecular interactions are novel and may serve as potential biomarkers of TB and LTBI, facilitating the development for a more sensitive, efficient, and cost-effective diagnostic assay for TB and LTBI for the Taiwanese population.

## 1. Introduction

Tuberculosis (TB) is an infectious disease usually caused by* Mycobacterium tuberculosis* (*Mtb*) [1]. Approximately one-third of the world's population is estimated to be latently infected with* Mtb* [2]. In other words, the host immune mechanism can sometimes keep the extent of the bacterial attack arrested at latent TB infection (LTBI). When the host's immune system becomes weakened, LTBI can progress to active pulmonary, or in fewer cases, extrapulmonary TB [3]. In fact, about 90% of those infected with* Mtb* are asymptomatic, showing signs of LTBI, with a 10% lifetime chance of developing active TB [4].

In primary active TB, the bacteria overcome the immune system defense and begin to multiply soon after the initial infection [1]. However, in LTBI, the bacteria remain dormant for many years before progressing to active TB. Even after treatment, there is still the risk of reactivation due to immunosuppression, or multiple-drug resistant TB bacteria [5].

Despite the advancement in pulmonary medicine, TB remains a significant global health issue. The only currently available vaccine is bacillus Calmette-Guérin (BCG), which shows decreased effectiveness after about ten years [5]. The tuberculin skin test (TST) and the interferon-gamma release assays (IGRA) are the usual clinical method for the diagnosis of TB and LTBI, with the latter being regarded as the more sensitive assay which measures the amount of interferon-gamma (IFN-*γ*) released by blood cells in response to specific* Mtb* antigens [6]. However, these methods often produce false positive results. Thus, developing a sensitive and efficient method for the detection of LTBI and understanding the disease pathology of TB represent a major challenge in the prevention of the disease.

To date, several studies have compared the gene expression profiles between healthy individuals and active TB or LTBI patients [7–10]. These findings reveal important transcriptionally regulated markers of key biological processes, including genes involved in inflammatory responses, immune defense, cell activation, homeostatic processes, and regulation of cell proliferation and apoptosis. It appears that TB and LTBI share similar affected pathways, in which specific molecular markers may be able to discriminate the two disease statuses.

More recent evidence suggests the use of microRNAs as biomarkers for active TB. MicroRNAs (miRNAs) are small, noncoding, single-stranded RNAs that modulate the expression of genes involved in development, cell differentiation, proliferation, and apoptosis [11]. It is estimated that as many as 20% of all human transcripts are targeted by microRNAs [12]. These tiny RNA molecules, proven to be more stable than messenger RNAs [13], actively circulate in bodily fluids and, thus, are thought to represent a more direct indicator of altered physiology [14]. Indeed, the therapeutic and diagnostic potentials of microRNAs have been the target of extensive study [15], especially in cancer research [16, 17].

In fact, microRNAs have been implicated to play important roles in the disease mechanisms of various infectious diseases. For example, the mouse microRNA, mmu-miR-29, has been shown to target IFN-*γ* and suppress immune responses against intracellular pathogens [18]. The human microRNA hsa-miR-32 has been found to modulate retrovirus PFV-1 replication [19]. Moreover, specific microRNA-gene interactions appeared to regulate the pathogenesis of HIV-1 infection [20].

Recently, through the use of expression array technology to explore the transcriptome on a global scale, several groups have investigated the possibility of using microRNAs or specific microRNA-gene associations as biomarkers for the diagnosis of TB or the differentiation between active TB and LTBI. These gene and microRNA expression studies identified candidate genes and microRNAs involved in cytokine and chemokine responses, inflammation, and intracellular trafficking in the progression from latent infection to active TB [7, 21–24]. Unfortunately, few of these findings are consistent with each other. Many of the discovered molecular markers vary due to diverse genetic background of the study population, differences in the study design, and the inherent complexity of the disease process.

With the latest advances in technology and bioinformatics, we believe utilizing complementary platforms to examine the differences in transcriptome between TB and LTBI in the Taiwanese population will help uncover novel biomarkers and build upon the knowledge regarding the disease diagnosis and pathology. Here, we present a systematic approach of combining gene and microRNA expression profiling to uncover the complex networks of molecular interactions associated with TB and LTBI. Our study began with the analysis of gene and microRNA expression profiles among active TB, LTBI, and healthy individuals. Candidate genes and microRNAs that appeared to be inversely correlated in expression were subsequently selected for a series of bioinformatics analyses to determine the nature of their relationships. Based on the computational predictions, a comprehensive microRNA-gene interaction network was constructed, revealing previously validated and novel molecular signatures that help improve our understanding and the diagnostic differentiation of TB and LTBI.

## 2. Materials and Methods

**Figure 1:**
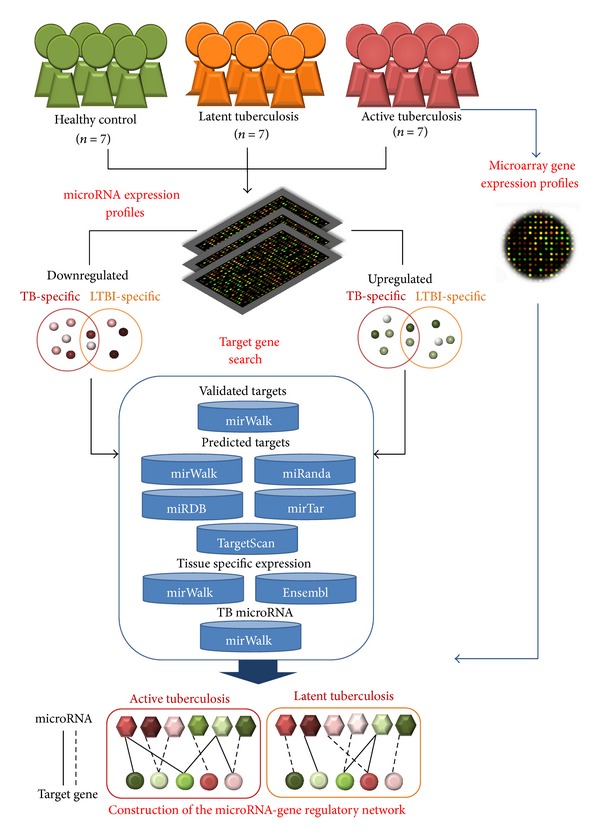
System flow of our analysis.

The analytical flow of the present study is illustrated in Figure 1. Profiling of microRNA and gene expression was performed to identify differentially expressed transcripts among 7 healthy control, 7 active TB, and 7 LTBI individuals. Differentially expressed candidates were categorized into up- and downregulated genes and microRNAs, and divided into three groups: TB versus healthy control, LTBI versus healthy control, and LTBI versus TB. Putative microRNAs targeting the differentially expressed transcripts were predicted using six microRNA knowledge bases.

### 2.1. Clinical Sample Collection

All procedures were reviewed and approved by the Institutional Review Board of Taoyuan General Hospital, Ministry of Health and Welfare, Taoyuan, Taiwan. Written informed consents were obtained from all participants. Eligibility for entry into the study was based on clinical signs and symptoms of* Mtb* infection. LTBI subjects were recruited from close contact with active TB patients, with positive T-SPOT TB test and negative chest radiograph, but without clinical evidence of active TB. Healthy controls were individuals who had not been in close contact with TB or LTBI patients and showed no clinical signs of TB or LTBI. Individuals with allergic diseases, diabetes, cancer, immune-compromised conditions, and coinfections with any types of infectious diseases were excluded. In total, seven healthy individuals, seven patients with active TB, and seven subjects with LTBI were included in the present study.

### 2.2. RNA Isolation

RNA was isolated from peripheral blood mononuclear cells. RNA quality was determined by an optical density (OD) 260/280 ratio ≥ 1.8 and OD 260/230 ratio ≥ 1.5 on a spectrophotometer and by the intensity of the 18 S and 28 S rRNA bands on a 1% formaldehyde-agarose gel. RNA quantity was detected by a spectrophotometer. RNA integrity was examined on an Agilent Bioanalyzer. RNA with a RNA integrity number (RIN) ≥ 6.0 and 28 S/18 S > 0.7 was subjected to microarray analysis.

### 2.3. MicroRNA and Gene Expression Analysis

RNA samples were subjected to Human OneArray v6 and Human microRNA OneArray v5 (Phalanx Biotech, Hsinchu, Taiwan). Data were analyzed with Rosetta Resolver System software (Rosetta Biosoftware, USA). Standard selection criteria to identify differentially expressed genes were (1) absolute log_2_ fold change ≥1; (2) false discovery rate of <0.05; (3) the intensity difference between two samples under comparison ≥1000; (4) individual intensity ≥500. Genes and microRNAs showing significant differential expression were categorized into TB versus healthy control, LTBI versus healthy control, and LTBI versus TB.

### 2.4. Bioinformatics Analysis

The candidate microRNAs were analyzed for associations with the candidate genes to reveal potential microRNA-gene interactions, in which decreased microRNA expression may be correlated with increased target gene expression and vice versa. In order to establish an* in silico* correlation between the microRNA profile in the blood and lung tissues, we input our list of differentially expressed microRNAs on miRWalk [25], a database that curates published results on experimentally verified microRNA tissue specificity, target genes, and disease associations. Next, for the selected microRNA candidates that have been shown to be active in both blood and lungs, a list of validated target genes was obtained from miRWalk [25]. For microRNAs with no validated target genes, target gene predictions were obtained by comparing among six microRNA knowledge bases, including miRWalk [25], miRTar [26], miRDB [27], miRANDA [28], RNA22 [29], and TargetScan [30], so as to take advantage of the strengths of each microRNA target prediction algorithm. A gene would be included in the target gene list if four out of these six databases generated the same prediction. The validated and predicted genes were then compared with differentially expressed microRNA-induced upregulated and downregulated genes in TB and LTBI. For matched genes, their tissues-specific expression profiles were determined through a search on the Ensembl system [31]. Only those target genes expressed in both blood and lungs were selected. Candidate microRNAs that have been implicated in TB were also determined through a search with the disease target tool in miRWalk [25].

### 2.5. MicroRNA-Gene Interaction Analysis

STRING (v9.1) [32, 33] was utilized to determine any known and putative interactions among the differentially expressed genes that were also estimated to be targeted by the differentially expressed microRNAs. The predicted relationships were based on a confidence level of 0.7, coexpression evidence, experimental validation, or database detection. To visualize the relationships among these TB- and LTBI-specific molecular signatures, a potential interaction network incorporating expression information was built with Cytoscape v3.1.0 [34]. In addition, genes targeted by TB-related microRNAs were mapped to KEGG pathways [35] by the gene set enrichment function in miRTar [26].

## 3. Results

Between LTBI and TB, 172 genes and 10 microRNAs presented significant differences in expression (Table 1). Additionally, there were 11 upregulated and 111 downregulated genes, plus one downregulated microRNA between TB and healthy controls. In contrast, 31 genes and 16 microRNAs showed increased expression, while 267 genes as well as six microRNAs exhibited decreased expression in LTBI compared to healthy individuals.

By using the validated target organ search in miRWalk [25], 11 out of the 30 differentially expressed microRNAs were found to target both blood and lungs. We hypothesize that these microRNAs represent potential correlates between the molecular profiles between the two tissues. Therefore, subsequent analyses were focused on these candidate microRNAs.

To uncover the potential microRNA-mediated regulation underlying the gene expression differences between TB and LTB, we utilized six microRNA target prediction databases and cross-validated the results to find putative microRNA-gene interactions (Table 2). Disease target search in miRWalk [25] indicated that some of these microRNAs have been implicated in other TB studies. However, our study also identified some novel microRNA-gene interactions that exhibited differential expression among TB, LTBI, and healthy controls.

**Figure 2:**
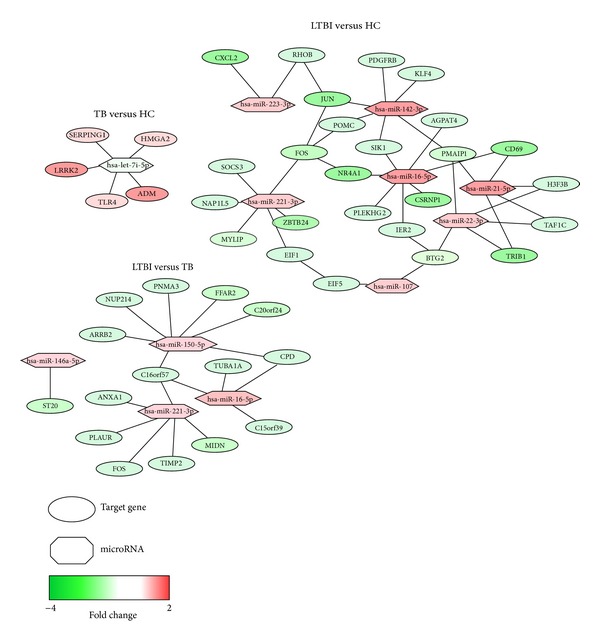
Potential TB- and LTBI-differentiating microRNA-gene interaction network. Color intensity indicates the expression of each molecule. Red indicates upregulated expression and green indicates downregulated expression.

The STRING [32, 33] network mapping tool was also employed to identify coexpressing or experimentally validated relationships among the candidate genes. The systematic prediction results based on validated and predicted gene, tissue, and disease targets were used to link the microRNAs and target genes in an interaction network (Figure 2). TB- and LTBI-specific differentially expressed microRNAs and genes may be involved in complex and dynamic interactions. The integration of the differential expression patterns in this graphical representation revealed the potential regulatory relationships among these molecular signatures.

**Figure 3:**
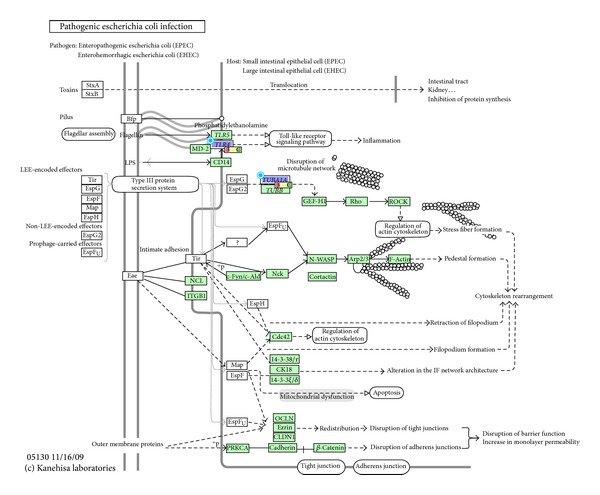
Interactions between hsa-miR-16-5p and* TUBA1A*, and hsa-let-7i-5p and* TLR4* that are predicted to be involved in the host response against pathogen infection.

Finally, by performing a disease target search, we identified candidate microRNAs that have also been associated with TB in the literature (Table 2). These TB-related microRNAs may be important key molecules specific to the disease pathology. In particular, the association between hsa-miR-150-5p and *β*-arrestin 2 (*ARRB2*) gene was mapped to the chemokine signaling pathway. Interestingly, LTBI-specific upregulated hsa-miR-16-5p and TB-specific upregulated hsa-let-7i-5p both target the same pathogenic infection pathway, though via different genes (Figure 3). In addition, microRNA hsa-miR-221-3p, with its target* FOS* (FBJ murine osteosarcoma viral oncogene homolog), were predicted to be involved in MAPK, B-cell receptor, and T-cell receptor signaling pathways.

## 4. Discussion

In this study, we integrated the available gene and microRNA expression array technology and bioinformatics tools to investigate the possibility of uncovering molecular events indicative of TB and LTBI. Our system flow allowed us to assess the results of gene and microRNA expression profiling through a combination of computational prediction and validation with published data.

Some of the candidate microRNAs identified by other groups were confirmed in our analysis, though with some inconsistencies in expression pattern. For example, microRNA hsa-miR-146a-5p appeared to be downregulated in TB patients in Spinelli et al.'s study [36] but was found to be upregulated by Furci et al. [37]. As well, microRNA hsa-miR-223-3p showed reduced expression in TB in one study [24] but enhanced expression in another published finding [38]. Our analysis, however, revealed downregulated hsa-miR-223-3p expression specific to LTBI. Whereas Meng et al. observed a lower expression of hsa-miR-150-5p in LTBI compared with healthy controls [39], we found the microRNA being expressed at a higher level in LTBI relative to TB. On the other hand, consistent with [22, 40], we found the level of hsa-miR-142-3p and hsa-miR-21-5p expression to be enhanced in LTBI relative to TB, and hsa-let-7i-5p expression, increased in TB compared with healthy controls. The discrepancies among studies emphasize the influence of genetic background and experimental design on the study results, underscoring the difficulty of deciphering the molecular mechanisms underlying TB pathology.

Yet, the most interesting inconsistency between our results and other studies is probably the expression pattern of hsa-miR-223 among active TB, LTBI, and healthy subjects. While Wang et al. [23] observed enhanced expression of hsa-miR-223 in TB patients versus nonactive TB group, the same microRNA appeared to be most abundant in healthy individuals, though the latter group was able to successfully validate their observation in a murine model. We, on the other hand, found this microRNA to be expressed at a higher level in individuals latently infected with TB.

Nevertheless, similar to Dorhoi et al.'s results [38], our computational prediction, coupled with the gene expression array data, showed an inverse correlation in expression patterns between hsa-miR-223 and its target gene* CXCL2*, a chemokine, that is, synthesized to facilitate an inflammatory response after injuries [41]. Deletion of miR-223 resulted in increased susceptibility to TB infection in mice and significantly augmented CXCL2 production, but upon neutralization of CXCL2, the severity of TB infection could be slightly reversed [38]. Thus, it appears that hsa-miR-223, as well as its modulation of CXCL2 abundance, may be able to determine, at least in part, the chance that an individual would succumb to a full-blown TB infection. This makes sense in the context of our finding, in which LTBI individuals possessed a higher level of hsa-miR-223 expression. It is likely that hsa-miR-223 transcription is induced upon the initial* Mtb* attack to arrest the infection at a latent state in individuals with LTBI.

In addition, we identified a potential interaction between hsa-miR-150-5p and the* ARBB2* (*β*-arrestin 2) gene, an immune regulator involved in cell adhesion, migration, and proliferation [42]. In particular, ARBB2 modulates the activity of G protein-coupled receptors to facilitate downstream inflammatory and immune responses [42]. The role that ARBB2 plays in the immune system is evidenced by its association with toll-like receptors, cytokines, chemokines, and various cell cycle and cell stress regulating signaling pathways in diseases such as rheumatoid arthritis, endotoxemia, sepsis, asthma, multiple sclerosis, and atherosclerosis [43]. Moreover, differential expression of* ARBB2* has been observed in tuberculosis [44, 45] and reduced expression of* ARBB2* has been correlated with an augmented level of IFN-*γ* [46], underscoring the importance of ARBB2 in the regulation of immune response. In our study, LTBI-specific downregulated* ARBB2* gene is the predicted target of the LTBI-specific upregulated hsa-miR-150-5p. The inverse relationship may indicate that this particular interaction plays a regulatory role in the immune response against* Mtb* bacteria.

Furthermore, we have also uncovered novel microRNA-gene interactions that may regulate the disease progression from latent to active TB. For example, in LTBI, both hsa-mir-16-5p and hsa-mir-221-3p appeared to be significantly upregulated compared with TB and healthy control. The microRNA hsa-mir-16 is a known inducer of apoptosis [47]. Our miRNA-gene interaction network revealed* TUBA1A* (tubulin alpha-1A) as a novel target gene that may modulate the host response to pathogenic infections through the interaction with hsa-mir-16-5p. Interestingly, TB-specific downregulated microRNA hsa-let-7i and its target gene* TLR4*, a toll-like receptor involved in innate immunity [48], were also mapped to the same pathway. Moreover, the potential interaction between hsa-miR-221-3p and* FOS* was linked to the T-cell and B-cell receptor signaling pathways. The* FOS* gene regulates various important biological processes such as cell proliferation, differentiation, and survival [49]. The potential interaction between the differentially expressed microRNAs and their target genes plays important roles in innate and adaptive immune responses in TB pathology. They may be used as molecular identifiers indicative of the two infection states.

In conclusion, we have performed a complementary analysis of gene and microRNA expression profiling and established a comprehensive microRNA-gene interaction network that may help differentiate between TB and LTBI. Note that our work is limited by the small sample size, and therefore the biomarkers may be specific to this particular study group. However, the strength of our analysis lies in the integration of various publicly available computational tools and experimental resources, allowing us to identify the most promising microRNA-gene associations through* in silico* predictions, gene expression profiling data, and published findings. Further, in hope to identify microRNA-gene interactions in the blood that would represent specific physiological conditions in the lungs, we filtered the candidate microRNAs and the corresponding target genes based on the similarity of their regulatory relationships in these two tissues.

Our work built on the emerging evidence that microRNA-gene interactions can be used as useful clinical biomarkers. Moreover, we have also uncovered novel TB and LTBI biomarkers specific to the Taiwanese population. These new molecular signatures are based on microRNA-gene interactions that may reflect the differences in TB disease state, further our understanding of TB pathogenesis, and facilitate the development of a molecular diagnostic platform for LTBI detection.

## Supplementary Material

Table S1. Differentially expressed blood-and lung-targeting microRNAs and their target genes in active TB versus healthy control (absolute fold change>1, FDR<0.05).
Table S2. Differentially expressed blood-and lung-targeting microRNAs and their target genes in LTBI versus healthy control (absolute fold change>1, FDR<0.05).
Table S3. Differentially expressed blood-and lung-targeting microRNAs and their target genes in LTBI versus active TB (absolute fold change>1, FDR<0.05).

## Figures and Tables

**Table 1:** Number of differentially expressed genes and microRNAs among TB, LTBI, and healthy controls.

Comparison	Upregulated	Downregulated
Genes		
TB versus control	16	111
LTBI versus control	31	267
LTBI versus TB	105	67
mircroRNA		
TB versus control	0	1
LTBI versus control	16	6
LTBI versus TB	9	1

Absolute fold change ≥ 1, FDR < 0.05.

**Table 2:** Selected blood- and lung-targeting microRNAs and their validated or predicted target genes.

microRNA	Target genes
TB versus control downregulated	
hsa-let-7i-5p	*ADM*, *HMGA2*, *LRRK2*, *SERPING1*, *TLR4 *
LTBI versus control upregulated	
hsa-miR-107	*BTG2, EIF5 *
hsa-miR-142-3p^a,b^	*KLF4, POMC, JUN, PMAIP1, PDGFRB, SIK1 *
hsa-miR-16-5p	*SIK1, NR4A1, IER2, AGPAT4, CSRNP1, PLEKHG2, CD69 *
hsa-miR-21-5p^b^	*CD69, H3F3B, TAF1C, TRIB1, PMAIP1 *
hsa-miR-22-3p	*H3F3B, TAF1C, TRIB1, PMAIP1, BTG2 *
hsa-miR-221-3p	*FOS, ZBTB24, EIF1, MYLIP, NAP1L5, SOCS3 *
hsa-miR-223-3p^b^	*RHOB*,* CXCL2 *
LTBI versus TB upregulated	
hsa-miR-146a-5p^b^	*ST20 *
hsa-miR-150-5p^b^	*CPD, ARRB2, FFAR2, NUP214, PNMA3, C20orf24, C16orf57 *
hsa-miR-16-5p	*CPD, C15orf39, C16orf57, TUBA1A *
hsa-miR-221-3p	*ANXA1, FOS, PLAUR, TIMP2, C16orf57, MIDN *

^a^validated microRNA-target interaction; ^b^indicates that these microRNAs have been implicated in tuberculosis.
