# Supplementary material for: Systematic Expression Profiling Analysis Identifies Specific MicroRNA-Gene Interactions that May Differentiate between Active and Latent Tuberculosis Infection
Source: Biomed Res Int. 2014 Sep 4;2014:895179. doi: 10.1155/2014/895179 (PMC4167957; doi:10.1155/2014/895179)
Supplement: Supplementary file 1 — Table S1. Differentially expressed blood-and lung-targeting microRNAs and their target genes in active TB versus healthy control (absolute fold change>1, FDR<0.05). Table S2. Differentially expressed blood-and lung-targeting microRNAs and their target genes in LTBI versus healthy control (absolute fold change>1, FDR<0.05). Table S3. Differentially expressed blood-and lung-targeting microRNAs and their target genes in LTBI versus active TB (absolute fold change>1, FDR<0.05). [file 895179.f1.pdf]

Supplementary Tables

Table S1. Differentially expressed blood-and lung-targeting microRNAs and their target genes in active TB versus healthy control (absolute fold change≥1, FDR<0.05)

**microRNA**	**Fold change**	**Target gene**	**Fold change**
hsa-let-7i-5p	-1.022620499	ADM	1.991955549
		HMGA2	1.083876589
		LRRK2	1.088987946
		SERPING1	1.921185639
		TLR4	1.109764875

Table S2. Differentially expressed blood-and lung-targeting microRNAs and their target genes in LTBI versus healthy control (absolute fold change≥1, FDR<0.05)

**microRNA**	**Fold change**	**Target gene**	**Fold change**
hsa-miR-107	1.087456866	BTG2	-1.254837843
		EIF5	-1.283869908
hsa-miR-142-3p	1.911618592	KLF4	-1.07784215
		POMC	-1.077178173
		JUN	-2.550017008
		PMAIP1	-1.349293986
		PDGFRB	-1.180991308
		SIK1	-1.112539392
hsa-miR-16-5p	1.807627123	SIK1	-1.112539392
		NR4A1	-2.07041723

		IER2	-1.007319135
		AGPAT4	-1.025013485
		CSRNP1	-3.09693761
		PLEKHG2	-1.040483174
		CD69	-2.02909225
hsa-miR-21-5p	1.700265107	CD69	-2.02909225
		H3F3B	-1.132305713
		TAF1C	-1.024709087
		TRIB1	-1.814667606
		PMAIP1	-1.349293986
hsa-miR-22-3p	1.137733536	H3F3B	-1.132305713
		TAF1C	-1.024709087
		TRIB1	-1.814667606
		PMAIP1	-1.349293986
		BTG2	-1.254837843

Table S2 continued.

**microRNA**	**Fold change**	**Target gene**	**Fold change**
hsa-miR-221-3p	1.266766297	FOS	-1.773572786
		ZBTB24	-1.78372674
		EIF1	-1.122163642
		MYLIP	-1.470759651
		NAP1L5	-1.027799939

		SOCS3	-1.097718601
hsa-miR-223-3p	1.344681533	RHOB	-1.347201513
		CXCL2	-3.698947401

Table S3. Differentially expressed blood-and lung-targeting microRNAs and their target genes in LTBI versus active TB (absolute fold change≥1, FDR<0.05)

**microRNA**	**Fold change**	**Target gene**	**Fold change**
hsa-miR-146a-5p	1.075774362	ST20	-1.57107693
hsa-miR-150-5p	1.074583994	CPD	-1.078308012
		ARRB2	-1.053042331
		FFAR2	-1.584404836
		NUP214	-1.357254667
		PNMA3	-1.096483529
		C20orf24	-1.337640838
		C16orf57	-1.070816645
hsa-miR-16-5p	1.396008554	CPD	-1.078308012
		C15orf39	-1.216084631
		C16orf57	-1.070816645
		TUBA1A	-1.113928236
hsa-miR-221-3p	1.109021	ANXA1	-1.042852811
		FOS	-1.274224394
		PLAUR	-1.022310972

	TIMP2	-1.169443261
	C16orf57	-1.070816645
	MIDN	-1.495474526
